# Primary plasma cell leukemia: consensus definition by the International Myeloma Working Group according to peripheral blood plasma cell percentage

**DOI:** 10.1038/s41408-021-00587-0

**Published:** 2021-12-02

**Authors:** Carlos Fernández de Larrea, Robert Kyle, Laura Rosiñol, Bruno Paiva, Monika Engelhardt, Saad Usmani, Jo Caers, Wilson Gonsalves, Fredrik Schjesvold, Giampaolo Merlini, Suzanne Lentzch, Enrique Ocio, Laurent Garderet, Philippe Moreau, Pieter Sonneveld, Ashraf Badros, Gösta Gahrton, Hartmut Goldschmidt, Sascha Tuchman, Hermann Einsele, Brian Durie, Baldeep Wirk, Pellegrino Musto, Patrick Hayden, Martin Kaiser, Jesús San Miguel, Joan Bladé, S. Vincent Rajkumar, Maria Victoria Mateos

**Affiliations:** 1 Amyloidosis and Myeloma Unit, Department of Hematology, Hospital Clínic of Barcelona, Institut d’Investigacions Biomèdiques August Pi i Sunyer (IDIBAPS), Barcelona, Spain; 2 Internal Medicine, Division of Hematology, Mayo Clinic, Rochester, MN USA; 3 Centro de Investigacion Medica Aplicada Instituto de Investigacion Sanitaria de Navarra, Clínica Universidad de Navarra, Pamplona, Spain; 4 Medical Department, Hematology, Oncology & Stem Cell Transplantation, University Medical Center Freiburg, Freiburg, Germany; 5 Hematogic Oncology and Blood Disorders, Levine Cancer Institute, Charlotte, NC USA; 6 Department of Hematology, CHU de Liège, Liège, Belgium; 7 Oslo Myeloma Centre, Department of Hematology, Oslo University Hospital, Oslo, Norway; 8 Department of Molecular Medicine, University of Pavia, Pavia, Italy; 9 Department of Medicine, Columbia University, New York, NY USA; 10 Department of Hematology, Hospital Universitario de Salamanca-IBSAL, IBMCC (USAL-CSIC), Salamanca, Spain; 11 Service d’Hématologie et Thérapie Cellulaire, Hôpital Saint Antoine, Paris, France; 12 Department of Hematology, University Hospital Nantes, Nantes, France; 13 Department of Hematology, Erasmus Medical Center, ZuidHolland, The Netherlands; 14 Greenebaum Cancer Center, University of Maryland, Baltimore, MD USA; 15 Department of Medicine, Karolinska Institutet, Stockholm, Sweden; 16 Internal Medicine V, University Hospital Heidelberg, Heidelberg, Germany; 17 Lineberger Comprehensive Cancer Center, University of North Carolina, Chapel Hill, NC USA; 18 Department of Internal Medicine II, University Hospital Würzburg, Würzburg, Germany; 19 Hematology/Oncology, Samuel Oschin Comprehensive Cancer Institute, Cedars-Sinai 2 Medical Center, Los Angeles, CA USA; 20 Bone Marrow Transplant Program, Penn State Cancer Institute, Hershey, PA USA; 21 IRCCS-CROB, Referral Cancer Center of Basilicata, Vulture, Italy; 22 Department of Haematology, St. James’s Hospital, Dublin, Ireland; 23 Myeloma Molecular Therapy Group, The Institute of Cancer Research, London, UK

**Keywords:** Cell biology, Myeloma, Cancer genetics

## Abstract

Primary plasma cell leukemia (PCL) has a consistently ominous prognosis, even after progress in the last decades. PCL deserves a prompt identification to start the most effective treatment for this ultra-high-risk disease. The aim of this position paper is to revisit the diagnosis of PCL according to the presence of circulating plasma cells in patients otherwise meeting diagnostic criteria of multiple myeloma. We could identify two retrospective series where the question about what number of circulating plasma cells in peripheral blood should be used for defining PCL. The presence of ≥5% circulating plasma cells in patients with MM had a similar adverse prognostic impact as the previously defined PCL. Therefore, PCL should be defined by the presence of 5% or more circulating plasma cells in peripheral blood smears in patients otherwise diagnosed with symptomatic multiple myeloma.

## Introduction

The original diagnostic criteria of plasma cell leukemia (PCL) were established in 1974 by Kyle requiring both more than 20% circulating plasma cells and an absolute count greater than 2 × 10^9^/l plasma cells in peripheral blood [1–3]. These criteria have provided a framework to define this disease entity along with the associated worldwide poor clinical outcome [4, 5]. However, these criteria had not been prospectively evaluated. With them, approximately 1–2% of patients with MM had PCL at diagnosis [6, 7].

PCL has been classified as primary when it presents “de novo” in patients with no evidence of previous multiple myeloma (MM) and as secondary when it is observed as a leukemic transformation of relapsed or refractory disease in patients with previously recognized MM. Thus, 60–70% of all PCL cases would be primary and the remaining 30–40% secondary [8].

In 2013, the International Myeloma Working Group consensus alerted for the first time that the criteria developed by Kyle, requiring both more than 20% circulating plasma cells and an absolute count greater than 2 × 10^9^/l plasma cells in peripheral blood, could be too restrictive [9]. One of the main comments was if probably only one of these criteria should be enough for the diagnosis of this entity. In fact, in many series, the presence of only one of these two criteria was considered sufficient for the diagnosis of PCL [4, 10, 11]. Moreover, patients with poor bone marrow reserve have baseline leukopenia and may not meet absolute criteria but may fulfill percentage criteria. In addition, a low proportion of plasma cells can be detected in peripheral blood in patients within the entire spectrum of plasma cell dyscrasias, including newly diagnosed MM, smoldering MM, and MGUS [12–14].

The consistently ominous prognosis, even after improving in the last decades, deserves a prompt identification of these patients to establish the most effective treatment for this ultra-high-risk disease [9]. However, the percentage of plasma cells is a continuous variable and the cut off should be based in retrospectively evaluating studies that have analyzed the prognosis of these patients.

With all the above considerations, we believe that the diagnostic criteria for primary PCL should be revisited. What number of circulating plasma cells in peripheral blood should be used for defining PCL?; there is no scientific rationale to establish the appropriate number of circulating plasma cells in peripheral blood for defining PCL and the percentage should be obtained from clinical evidence. Over the last few years, some retrospective studies have been conducted and published by different groups [15–17]. The aim of this position paper is to revisit the diagnosis of primary PCL according to the presence of circulating plasma cells in patients otherwise meeting diagnostic criteria of symptomatic MM.

## Methods

We have reviewed the literature, looking for validation of alternative cutoffs for the diagnosis of primary PCL in prospective or retrospective series of patients with MM. We could identify only two retrospective series where the question about what number of circulating plasma cells in peripheral blood should be used for defining PCL [16, 17]. The objective in both groups was to investigate if lower values (such as 5% or more plasma cells) have the same prognostic impact as the historical criteria. In a previous report from China, the impact of the morphological examination was also assessed, even if the cutoff for high-risk group/PCL was not consistent with the other reports [15]. This study was excluded because it only stratified MM patients with vs. without circulating plasma cells using a cut-off of 2%, instead of 5% as in the other included papers. There are other experiences also validating the lower cutoff of 5% with prognosis significance similar to classical PCL but only presented at meetings [18, 19].

The objective was to summarize the results of previously published data regarding the outcomes of patients with a lower circulating plasma cell threshold from both series, one from 5 university hospitals in Catalonia, Spain (100 patients) [16] and the other from Mayo Clinic (176 patients) [17], in order to support the new definition of PCL. These cases were compared to simultaneous and consecutive controls over the same period of time in the first series (382 patients) and historical controls in the second (9724 patients), respectively. The Catalan series was published previously, serving the second one (Mayo Clinic) as validation series.

Peripheral blood smears stained with Wright–Giemsa were used for calculating plasma cell percentage by morphology: patients with no peripheral blood smears were excluded in both series. In the Mayo Clinic series, the percentage was obtained from the peripheral blood count showed in the medical report. The number of evaluated cells was not specified. In addition, in the Catalan series 5 expert hematologists on peripheral blood cytology reviewed all the smears, with a minimum of 100 nucleated cells per smear systematically counted using the same common criteria. Overall survival (OS) was the primary outcome and was measured from the date of diagnosis to the date of death or last follow-up in both studies. OS and baseline characteristics of these patients were compared to the controls and the independent prognostic impact of circulating plasma cells was addressed.

In both studies, the primary outcome was OS, which was defined as the duration between date of diagnosis and death, or censored at last follow-up. Survival analysis was performed using the Kaplan–Meier method and differences were tested for statistical significance using the log–rank test. Multivariate analysis was conducted using the Cox proportional hazards model in the Catalan study.

## Results

### Patient characteristics

As previously stated, there are two recent publications [16, 17] that have addressed this issue in Europe and the US (Table 1).

**Table 1:** Main results of the two clinical studies used for this position paper [16, 17].

	Clinical series	
Variable	Granell et al. [16]	Ravi et al. [17]
Number of patients	482^a^	176^b^
Age (median; years)	69	62
Sex (M/F); %	42/58	56/44
Patients with CPC; *n*	100	176
*Distribution according CPC (n; %)*^c^		
1–4%	83 (83%)	54 (31%)
5–20%	12 (12%)	63 (36%)
More than 20%	5 (5%)	59 (34%)
*Median overall survival according to CPC; months*		
0%	47	53
1-4%	50	17
5–20%	6	13
More than 20%	14	13

^a^Totally, 382 patients (included in the number in the table) without circulating plasma cells were used as controls.

^b^Totally, 9724 patients (not included in the number in the table) diagnosed in the same period without circulating plasma cells at diagnosis were used as controls.

^c^Considering only patients with circulating plasma cells.

*CPC* circulating plasma cells.

First, a series of 482 patients from Catalonia (Catalan Myeloma Group), Spain, between 2008 and 2013 with newly diagnosed MM or PCL was reported [16]. Patients were classified into 4 categories according to the percentage of circulating plasma cells: 0% (382; 79.2%), 1–4% (83; 17.2%), 5–20% (12; 2.5%), and the conventional definition of PCL (more than 20%) (5; 1%). Hundres patients (20.7%) showed at least 1% of circulating plasma cells. Considering only the 100 patients with circulating plasma cells in order to compare with the Mayo Clinic series, the percentages of circulating plasma cells would be: 1–4% (83%), 5–20% (12%), and more than 20% (5%). The 382 patients without circulating plasma cells were used as controls.

More recently, Ravi et al. from the Mayo Clinic [17] reviewed outcomes of 176 patients with MM who had circulating plasma cells at diagnosis between 1971 and 2006, to determine whether a lower threshold could be used to diagnose PCL. They classified the patients according to 3 categories, 1–4% (54; 31%), 5–19% (63; 36%) and ≥20% (59; 34%). The comparisons in this series were established with 9724 patients diagnosed in the same period without circulating plasma cells at diagnosis (controls).

### Clinical features of patients with 5% or more circulating plasma cells

In general, patients in the Catalan group were slightly older (median age 69 vs. 62 years) and with different sex distribution (M/F 42/58% vs. 56/44%) than in the Mayo Clinic series. Baseline characteristics of the different groups of patients according to the presence and number of circulating plasma cells were only evaluated in the Spanish study. Differences in age, sex, myeloma isotype, LDH, Durie/Salmon, and ISS stages were not statistically significant. However, patients with 5–20% had lower platelet counts (median 86 × 10e9/*L* vs. 214 × 10e6/*L*) and a higher proportion of bone marrow plasma cell infiltration (median 53% vs. 36%) than those with no circulating PC. The number of patients with available cytogenetic data was insufficient for accurate statistical calculations.

Treatment received was analyzed in the whole series in the Catalan group and only in the patients with circulating plasma cells in the US study, so the differences cannot be compared. However, for historical reasons, the more frequently used regimens in the Mayo series were cytotoxic chemotherapy without new agents (58%) while bortezomib-based schemes were used more frequently in the Catalan report (47.7%). Stem cell transplantation was more frequently reported in the Catalan series (32% vs. 20%).

### Outcome according to circulating plasma cell by morphology

Median OS according to the circulating plasma cell groups (0%, 1–4%, 5–20% more than 20%) was 47, 50, 6, and 14 months, respectively in the Spanish group. In the US series, median OS of the different groups (0%, 1–4%, 5–19%, and 20% or more) was 53, 17, 13, and 13 months (Table 1). Thus, in the Catalan study the presence of 1–4% CPCs does not influence OS (the same OS for patients without CPCs; 47 vs. 50 months), while in the US trial there was a major impact (53 vs. 17 months); in fact, the OS of these patients was not very different from that of patients with >5% CPCs (13 months). We have to note that in the Catalan study median follow-up was 28 months, which makes the median OS for patients <5% plasma cells an expected/projected measurement rather than an actual measurement. In the US study, the median follow-up was 6.8 years.

In both series, due to the similarities in the outcomes observed for patients with 5% or more circulating plasma cells, the two groups were stratified. In the Catalan study, survival of those with ≥5% CPCs was significantly shorter when compared with a cohort of MM patients who did not have circulating plasma cells at diagnosis or had 1–4% (1.1 vs. 4.4 years, RR 4; 95 CI: 2.1–7.3; *p* < 0.001). In the US series, also shorter survival was observed in the groups of patients with more than 5% CPCS (1.17 vs. 4.8 years; *p* < 0.001).

In the Catalan series, the significance was retained for those patients with 5–19% circulating plasma cells and treated with novel agents. Cytogenetics could not be incorporated in this series for lack of information. However, in the Mayo series, median OS in those with ≥5% of circulating plasma cells was significantly lower compared to patients with both standard and high-risk MM (1.4 vs. 7.5 vs. 4.3 years, respectively; *p* < 0.001).

A Cox-regression multivariate analysis was conducted in the Catalan study and the presence of 5–20% circulating plasma cells emerged as an independent prognostic feature predicting for a shorter OS (relative risk 4.9, 95% CI: 2.6–9.3) regardless of age, creatinine, Durie–Salmon system stage, and International Staging System (ISS) stage.

## Discussion

The correct and timely diagnosis of PCL is dependent upon the ability of the pathologist or hematologist to screen and recognize plasma cells in the peripheral blood smear. They should be aware of the clinical relevance of a careful morphological examination of peripheral blood smears to exclude the presence of circulating plasma cells [9].

PCL is unique in the spectrum of malignant monoclonal gammopathies and characterized by a poor prognosis, the shortest OS and the elevated frequency of some genetic abnormalities (i.e., deletion 17p, hypodiploidy, and *t*(11;14)) along with some distinctive molecular and epigenetic characteristics [9, 11, 20–22]. Some classical clinical features, reduced bone marrow reserve, extramedullary involvement, and elevated LDH, are more common than in patients with “standard” MM [6, 8, 9, 11, 23]. Short duration of response in primary PCL has been disappointing. Even with significant improvement in the last years [5], the use of immunomodulatory drugs, proteasome inhibitors and either autologous or allogeneic stem cell transplantation cannot overcome the poor prognosis in this disease [10, 20, 24–29].

In the two series analyzed [16, 17], the morphology examination was crucial to identify patients bearing clinical and prognostic features compatible with the described natural history of primary PCL. Consistently between both articles, the OS in patients with 5% or more circulating plasma cells was significantly shorter than in patients with MM without circulating plasma cells. This significance was retained in one of the studies in multivariate analysis [16]. Moreover, the prognosis was quite similar in both series with these patients and those with the conventional definition of PCL. At least in the US series, this adverse outcome was worse than patients with high-risk MM and less than 5% of circulating plasma cells, reflecting a different biology beyond standard cytogenetic prognosis [17]. Patients with ≥5% circulating plasma cells showed lower platelet count and higher bone marrow plasma cell infiltration, two clinical features of primary PCL [16]. In a previous study, the impact of the morphological examination was also assessed, even if the cutoff for high-risk group/PCL was not consistent with the other reports (2%) [15]. Several limitations of this consensus include that both retrospective series [16, 17] are not entirely and well comparable, as well as data in the literature about circulating plasma cells by morphology is rather scarce. There is no information about secondary PCL in this regard. The change in the criteria for diagnosis of PCL are, in principle, applicable only to primary PCL patients and the change into the definition for secondary PCL criteria requires further investigation.

Thus, the presence of ≥5% circulating plasma cells in patients with MM has a similar adverse prognostic impact as the previously defined PCL, increasing between 0.7 and 2.5% the number of patients who would be classified as primary PCL. We therefore propose that the definition of PCL should be revisited, and according to these two studies, all patients diagnosed with MM and with circulating plasma cells of ≥5% detected by morphology in the examination of the blood smear should be considered as primary PCL. Although there might be some limitations, especially because of the retrospective nature of the two studies, the revision of this new definition would increase the awareness for the diagnosis of PCL and subsequent treatment adaptation. In the Catalan study, many patients were not treated with relative new regimens; majority of patients in the US study were treated with proteasome inhibitor and immunomodulatory agent-based treatment. Despite this difference, both studies independently showed similar results with respect to OS. This observation also suggests that current treatment strategies could not be able of overcoming the adverse prognostic impact induced by having circulating PCL of ≥5%. However, a significant caveat of both studies is that the majority of included patients with circulating plasma cells were not treated with novel agents and/or autologous stem cell transplantation. It may be possible that the current standard of triplet induction regimen, autologous transplantation (for suitable patients) and maintenance (with dual agent, including a proteasome inhibitors) could overcome the dismal prognosis of a relative lower burden circulating plasma cells seen in these studies than in those with the previous definition of primary PCL.

There is an urgent need to prospectively investigate the molecular characteristics of PCL further, to gain a better understanding of the genomic mechanisms as a basis to develop optimal, stratified treatment approaches. Recent trial designs have demonstrated that addressing both ultra high-risk myeloma and PCL jointly can further overcome barriers to recruitment for these patient populations with high unmet need. We recommend removing PCL as routine exclusion criteria for MM trials. Patients with this new definition of primary PCL should be included in clinical trials and reported as high-risk feature, in order to not penalize the possibility of receiving new agents.

In any given patient, the presence of a few circulating plasma cells demonstrated by conventional morphology is still a marker for a highly proliferative and aggressive process [17]. Lower levels (more than 1 or 2% plasma cells in peripheral blood) could define a higher risk MM with prognosis very close to PCL [15, 17]. Patients with an ‘early’ PCL may rapidly progress; these patients must be followed closely because they may develop PCL in a very short time. However, it has yet to be demonstrated that aggressively treating patients with lower circulating plasma cells that previous definition of primary PCL will yield superior outcomes.

Additional methods to detect early primary PCL should be a high priority and warrant further studies. Thus, the use of slide-based immunofluorescence microscopy, multiparametric flow cytometry and next-generation flow for plasma cell quantification in peripheral blood has been associated with survival in MM [30–33]. These studies used more sensitive techniques than those in the two studies discussed. However, for each technique, the cutoff and the percentage of patients having PCL should be further explored and validated. Conventional cytology is a simple and inexpensive technique that can be applied in any clinical laboratory and although it is not able to identify clonality of circulating plasma cells, the new definition can be considered as a starting point for further investigation. The incapacity to establish clonality is a limitation in the rare case of polyclonal circulating plasma cells in some patients with MM [34, 35] and could be readily resolved with flow cytometry immunotyping [33].

Gathering all the available information and putting that into perspective, the impact of the presence of 5–20% plasma cells in peripheral blood in patients with MM has a similar ominous meaning as presence of ≥20% circulating plasma cells. The proposal is to establish a new diagnostic definition of this devastating disease by the International Myeloma Working Group. This new definition with morphologic criteria, using conventional staining techniques in peripheral blood, would increase up to three times the number of patients diagnosed with primary PCL. This strategy is available worldwide, with independence from economic and technological resources. Finally, these patients, otherwise classified as symptomatic MM, should be enrolled early into trials using the newest immunotherapies and combined strategies available in an attempt to improve survival.

### Consensus recommendation

Primary PCL is defined by the presence of 5% or more circulating plasma cells in peripheral blood smears in patients otherwise diagnosed with symptomatic MM. Careful examination of peripheral blood by conventional microscopy should be done in all patients with MM. A minimum of 100–200 nucleated cells per smear should be systematically analyzed by an experienced pathologist/hematologist. Patients with this new definition should not be excluded from clinical trials.
